# Laser‐Printed, Flexible Graphene Pressure Sensors

**DOI:** 10.1002/gch2.202000001

**Published:** 2020-03-11

**Authors:** Altynay Kaidarova, Nouf Alsharif, Barbara Nicoly M. Oliveira, Marco Marengo, Nathan R. Geraldi, Carlos M. Duarte, Jurgen Kosel

**Affiliations:** ^1^ Computer, Electrical and Mathematical Sciences and Engineering (CEMSE) Division King Abdullah University of Science and Technology (KAUST) Thuwal 23955 Kingdom of Saudi Arabia; ^2^ Red Sea Research Center (RSRC) and Computational Biosciences Research Center King Abdullah University of Science and Technology (KAUST) Thuwal 23955 Kingdom of Saudi Arabia

**Keywords:** flexible devices, graphene, laser printing, piezoresistive materials, pressure sensors, wearables

## Abstract

While the outstanding properties of graphene have attracted a lot of attention, one of the major bottlenecks of its widespread usage is its availability in large volumes. Laser printing graphene on polyimide films is an efficient single‐step fabrication process that can remedy this issue. A laser‐printed, flexible pressure sensor is developed utilizing the piezoresistive effect of 3D porous graphene. The pressure sensors performance can be easily adjusted via the geometrical parameters. They have a sensitivity in the range of 1.23 × 10^−3^ kPa and feature a high resolution with a detection limit of 10 Pa in combination with an extremely wide dynamic range of at least 20 MPa. They also provide excellent long‐term stability of at least 15 000 cycles. The biocompatibility of laser‐induced graphene is also evaluated by cytotoxicity assays and fluorescent staining, which show an insignificant drop in viability. Polymethyl methacrylate coating is particularly useful for underwater applications, protecting the sensors from biofouling and shunt currents, and enable operation at a depth of 2 km in highly saline Red Sea water. Due to its features, the sensors are a prime choice for multiple healthcare applications; for example, they are used for heart rate monitoring, plantar pressure measurements, and tactile sensing.

## Graphical abstract

A facile, one‐step laser printing process is used to fabricate a flexible graphene pressure sensor with a broad measurement range. As a wearable device, it is used for heart rate monitoring, plantar pressure measurements, and tactile sensing. For underwater operation, measurements are conducted at a depth of 2 km (≈20 MPa) in highly saline Red Sea water.

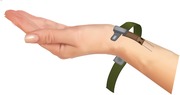

## 1 Introduction

Pressure sensors have been critical elements in many applications across various industries and many different types of sensors have been developed to satisfy the needs of specific applications. Recently, there is a growing demand for flexible and lightweight pressure sensors, requiring the development or utilization of new technologies.^[^ ^1^, ^2^, ^3^ ^]^ Extensive research has been undertaken to obtain robust pressure sensors for wearable devices,^[^ ^4^, ^5^ ^]^ human–machine interactions,^[^ ^6^ ^]^ environmental monitoring systems,^[^ ^7^ ^]^ and electronic and marine skin applications.^[^ ^8^ ^]^ In the past few years, pressure sensors based on nanostructured materials, including metal nanowires,^[^ ^9^ ^]^ carbon nanotubes,^[^ ^10^ ^]^ and ZnO nanowire arrays,^[^ ^11^ ^]^ have been explored based on a range of mechanism types such as capacitive, piezoelectric, triboelectric, and piezophototronic. The large‐scale fabrication of such pressure sensor devices using non‐traditional materials presents challenges. For example, piezoresistive sensors show advantages, such as simple device structure, large measurement range, easy read‐out mechanism, and potentially high pixel density.^[^ ^1^ ^]^ However, the sensitivity of most resistive sensors reduces considerably at higher pressures (>5 kPa), which can be inadequate for certain applications.^[^ ^1^ ^]^ Maintaining a high sensitivity over a wide pressure range is an important requirement for realizing reliable sensing systems.

Conventional pressure sensors are typically made of rigid materials and cannot conform to nonplanar surfaces.^[^ ^4^ ^]^ In case of flexible, bendable sensors, it is also important to maintain the pressure‐sensing ability during deformation on curved and nonplanar surfaces. Flexible sensors are relevant in applications for rollable touch displays, biomonitoring, and electronic skins.^[^ ^2^ ^]^ Despite extensive research and development in the field of pressure sensors, the fabrication process often requires multiple steps, high‐vacuum conditions, and time‐consuming syntheses.

Graphene has been recently identified as a promising material for sensing applications owing to its outstanding mechanical and electrical properties.^[^ ^12^ ^]^ However, the surfactants used during the fabrication are difficult to remove as is the production of graphene in pieces large enough for a practical purpose. There is also a reduction in the graphene sheet size due to the excessive sonication process, or requirements of a high boiling point and multi‐step synthesis routes, which consume valuable resources and make it one of the most expensive materials to manufacture.^[^ ^13^, ^14^ ^]^

In 2014, it was discovered that graphene could be synthesized via direct lasing of PI films, leading to the formation of porous graphene, by a photothermal process associated with the localized high temperature.^[^ ^15^ ^]^ This is a single‐step, highly tunable, and inexpensive process that does not need reducing agents, expensive equipment, solvents, and subsequent treatments or other supporting processes. Since then, this so‐called laser‐induced graphene (LIG) has been utilized to create numerous electronic devices in a single step.^[^ ^16^, ^17^, ^18^, ^19^, ^20^ ^]^ In the present study, we demonstrate the use of LIG to fabricate piezoresistive, mechanically flexible, lightweight, and robust pressure sensors with a large measurement range, thereby offering promise across the whole spectrum of demands for pressure sensors.

## 2 Experimental Section

### 2.1 Fabrication Process

**Figure 1:**
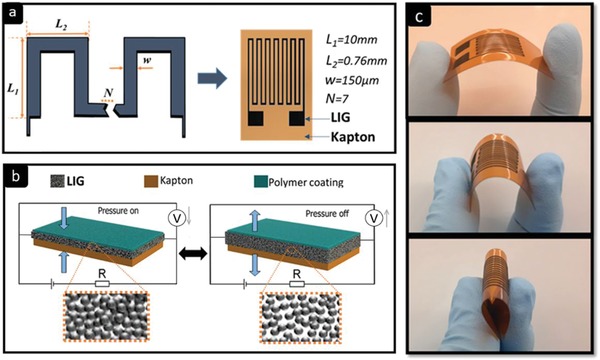
a) Design of LIG pressure sensor. b) Operation principle of pressure‐sensitive LIG sensors. c) Demonstration of the flexibility of LIG pressure sensors.

Conductive porous graphene electrodes were directly patterned on commercial PI films with the thickness of 125 µm (Kapton #IM301449, DuPont, Delaware, USA). The samples were processed in ambient air using a CO_2_ infrared laser (wavelength 10.6 µm, laser spot diameter ≈150 µm, Universal Laser Systems PLS6.75) that emitted linearly polarized continuous wave light with a power of up to 75 W. The optimized laser scanning speed and power to control the electrical conductivity were 3 cm s^–1^ and 5 W, respectively, 1000 pulses per inch and 3 mm laser‐to‐surface distance. The printed electrode pattern was structured in a meander shape in order to maximize the area of the LIG. The line segments were connected in series such that resistance variations in each segment could contribute accumulatively to provide a large change in resistance, while keeping the minimum electrode width (≈150 µm, corresponding to the laser spot diameter). The layout of the sensor with dimensions is shown in **Figure** **1**a. A stable electrical connection to the sensor was obtained by through holes of 120 µm in diameter, which were created using 30 W of power and 20 cm s^–1^ speed, and mechanically threading wires through the holes (Figure S1, Supporting Information). Finally, a protective polymethyl methacrylate (PMMA) coating was applied, which is necessary for applications in conductive media, and to avoid interference with the measurement, due to shunt currents. To this end, a 15 µm thick layer of PMMA (Kafrit, Inc.) was thermally laminated with a 4 min warm‐up time (P42DE‐WE, Atlas, Inc.).

### 2.2 Transduction Mechanism

The conductivity of the porous LIG material arises from a densely interconnected 3D network of graphene flakes. The mechanism behind LIG pressure sensors is based on transducing pressure stimuli into a variation of resistance. The overall resistance of the sensor can be approximated as the sum of two components

(1) $$R\sim R_{E}+R_{LIG}\sim R_{E}$$

where *R*_E_ is the resistance of the electrodes and *R*_LIG_ is the piezoresistive material. Since the employed copper electrodes are highly conductive (*R*_LIG_ >> *R*_E_), the pressure responses mainly result from changes in resistance of the LIG, which is introduced by cross‐sectional deformation under external pressure, as shown in Figure 1b. The pressure stimulus decreases the distance between neighboring interlayers of graphene, which consequently increases the contact area and reduces the resistance, as shown in the insets of Figure 1b. In other words, more graphene conductive paths are established, when the external force is applied, leading to a resistance decrease. LIG easily deforms due to the low Young modulus (≃40 kPa),^[^ ^21^ ^]^ and when the pressure is released, the porous LIG recovers to its initial state. The absolute change in resistance is tailored by the number of turns and dimensions of the meander‐shaped LIG electrodes. The resulting sensors are also highly flexible as illustrated in Figure 1c, where an LIG sensor is bent to various radii of curvature. It should be noted that when a relatively rigid material like PMMA (*E* ≈ 3.2 GPa) is laminated on top of the LIG‐polyimide stack, almost all the stress gets transferred to the LIG upon applying pressure, which provides a pathway for insulating and passivating the sensor.

## 3 Results and Discussion

### 3.1 Characterization of LIG Pressure Sensors

**Figure 2:**
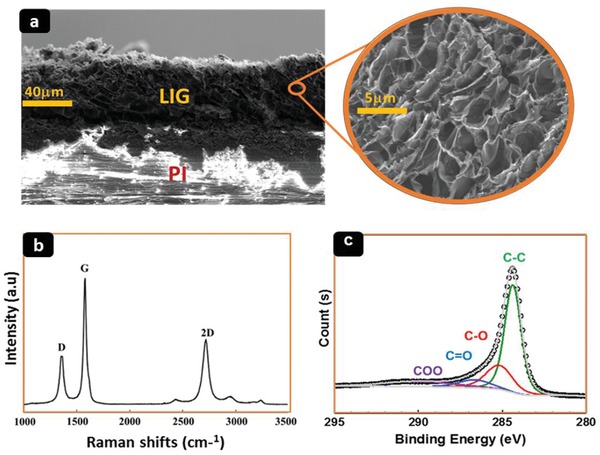
a) Cross‐sectional SEM images of porous graphene structures on PI after laser irradiation. The inset with a higher magnification shows randomly arranged and interconnected graphene flakes. b) Raman spectrum of LIG acquired with a laser wavelength of 473 nm. c) High‐resolution XPS spectrum of the C1s region of LIG.

The microstructure of the LIG sensors was analyzed using scanning electron microscopy (SEM, Quanta 600 FEG Systems), confocal Raman microscopes (Alpha300AR+, WITec), and X‐ray photoelectron spectroscopy (XPS, ESCA 3400, Amicus Kratos Analytical). **Figure** **2**a is the SEM image of an LIG cross section under low magnification, where the porous and carbonized structure of ≈40 µm thickness can be identified on top of the remaining PI. The SEM image of LIG under high magnification (inset) indicates that the entire volume of the LIG is composed of highly porous multilayer graphene flakes. The Raman spectrum of the LIG in Figure 2b presents three characteristic peaks of graphene: D peak at 1360 cm induced by defects or bent sp2 carbon bonds, G peak at 1580 cm related to graphite‐derived structures, and 2D peak at 2720 cm, which is the second harmonic of the D band and the main one in monolayer graphene that can arise from graphene structures induced by laser processing. This observation is consistent with results obtained from the XPS spectrum, shown in Figure 2c, which features the dominant C—C peak with significantly suppressed C—O, C=O, and COO peaks, suggesting the domination of sp2 carbons and breakage of these chemical bonds.

**Figure 3:**
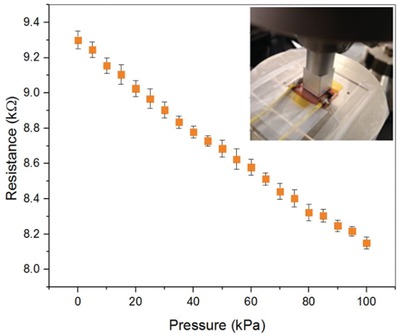
The average resistance of five measurements of a coated LIG pressure sensor as a result of consecutive linear loading cycles in an electromechanical pull tester. The error bars indicate the standard deviation.

**Figure 4:**
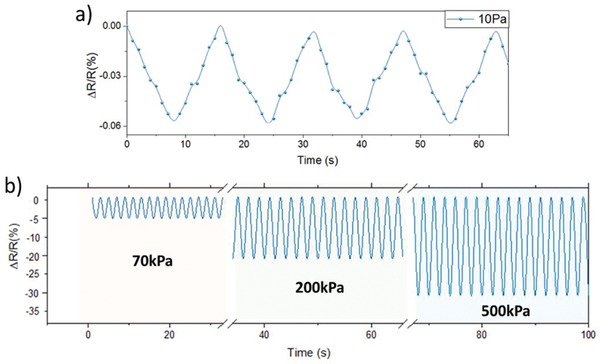
a) LIG sensor responses to low pressure. b) The operational stability of an LIG sensor at different pressures.

PMMA‐coated LIG pressure sensors were examined using static pressures in an electromechanical pull tester (5900‐Series, Instron, Inc.) by applying a rectangular press load of 1 kN at 1 s intervals. The sensitivity of a piezoresistive pressure sensor is defined^[^ ^2^ ^]^ as

(2) $$S=\frac{\Delta R\text{/}R}{p}$$

where *p* indicates the applied normal pressure and ∆*R*/*R* is the relative change in the resistance. A continuous current of 100 µA was applied with a Keithley 2400 Source Meter through the LIG pressure sensor (as in all consecutive experiments) to measure the change of electrical resistance during the compression of the sensor. **Figure** **3** shows the response of the sensor with an average linear sensitivity of 1.23 × 10^−3^ kPa and a standard deviation of σ ± 0.005 × 10^−3^ kPa . The performance of the sensor was then evaluated under low pressure regime using the sensor shown in Figure 1a. The lowest detectable pressure of 10 Pa resulted in a resistance change of 0.006%, as shown in **Figure** **4**a. The response can also be observed in the Movie S1, Supporting Information, where the LIG pressure sensor is used to switch on a red light‐emitting diode. Figure 4b shows that the sensor responds repeatedly over multiple sinusoidal load cycles with 70, 200, and 500 kPa. A 15 000 load cycle experiment (>100 h) is presented in Figure S2, Supporting Information, and shows no deterioration of the sensor response.

### 3.2 Biocompatibility

**Figure 5:**
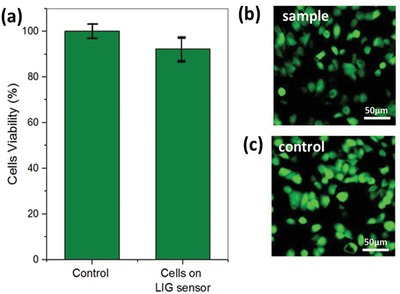
Biocompatibility assessment of the sensor. a) AlamarBlue assay and b,c) confocal images show the viability of HCT116 cells to grow on top of the sensor after 24 h. The control shows the viability of cells growing on a cover slide, while the sample shows viability of the cells growing on top of the sensor. Error bars represent the standard deviation of six replicates. *p* > 0.05.

The biocompatibility of the sensor was assessed using HCT 116 cells and two methods: coulometric assay (alamarBlue cell viability assay) and confocal microscopy. The alamarBlue assay was used to quantify the cell viability, while the LIVE/DEAD fluorescence staining method (calcein for live cells and ethidium homodimer‐1 for dead cells) was used to visualize the cell viability. The preparation methods of the samples are discussed in Supporting Information. The coulometric assay results are presented in **Figure** **5**a, showing a high biocompatibility of the LIG sensor by maintaining a high cell viability (>90%), which is not significantly different to the control after 24 h of incubation. In addition, the fluorescence staining method revealed the ability of HCT 116 cells to grow in a confluent way on the sensor (Figure 5b). Most of the cells on the sensor were calcein‐stained 24 h after growth, indicating high biocompatibility similar to the control (Figure 5c).

## 4 Applications

### 4.1 Underwater Pressure Monitoring

**Figure 6:**
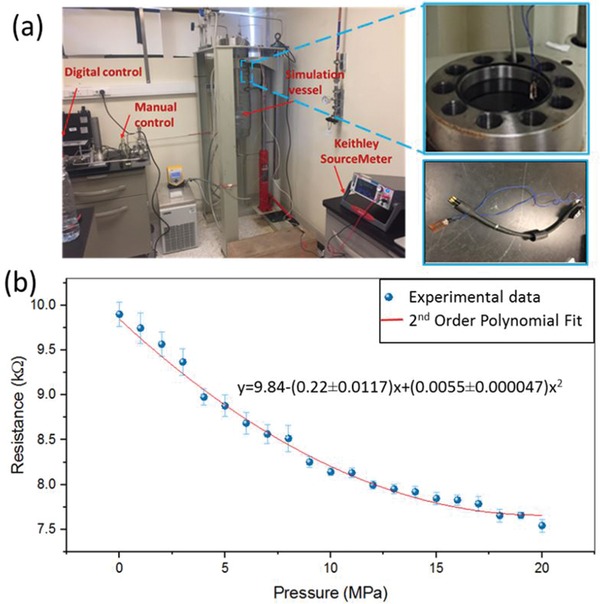
a) Experimental setup for the high‐pressure simulation and real‐time testing in the harsh seawater environment. b) The average resistance of five measurements of an LIG pressure sensor in the pressure vessel. The error bars indicate the standard deviation. The coefficient of determination (*R* ^2^) is 0.984.

To evaluate the sensors for high‐pressure underwater applications, they were placed inside of a pressure vessel (3755 PSI, OSECO) using Type T Hydraulic Deadweight Tester (DM‐TQ‐150‐1AL/C, AMETEK), which is the primary calibration standard tool for high‐pressure measurements. The metallic cylinder assembly of 60 cm in height (15 cm diameter) was filled with Red Sea water, and the sensor was placed inside the vessel connected with waterproof wires to the Keithley's Series 2400 SourceMeter, as shown in **Figure** **6**a. The response shows a hysteresis error, quantified as the maximum difference between the loading and unloading curve divided by the full‐scale output, of 2.9%, which is much lower than the hysteresis observed for conductive foams.^[^ ^22^, ^23^ ^]^ Figure S3, Supporting Information, shows the cross section of two LIG sensors before and after application of high pressures (20 MPa). The results suggest that there is no perceptible change or damage in the morphology of the LIG after applying high pressures and the possibility of using the sensor of at least to ≈2 km depth in the seawater.

### 4.2 Pulse Rate Monitoring

**Figure 7:**
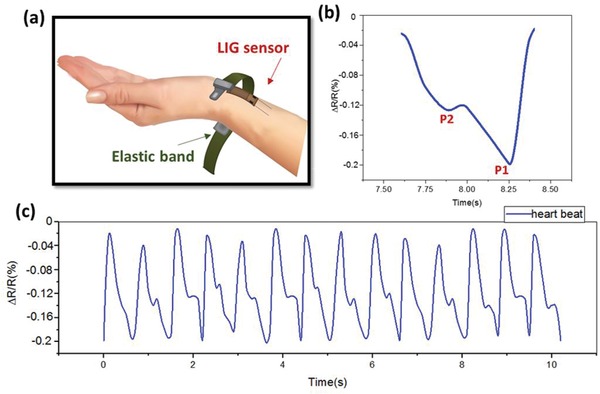
a) Schematic of the wrist LIG pressure sensor for heart rate monitoring on the radial artery. b) Signal of one pulse. P1 is the “novice wave” associated with cardiac shrinkage, while P2 is the “reflected wave” associated with the reflection from a peripheral blood artery. c) Measurements of the arterial pulse over 10 s.

Daily pulse monitoring could provide abundant physiological and pathological information of the human cardiovascular system, but currently popular optical methods have their limitations especially at faster heart rates.^[^ ^24^ ^]^ While optical methods have improved a lot, they still suffer from inaccuracy and frequent failure, due to the movement artifacts at the sensor–skin interface and the optical scatter from time‐varying motion (blood flow). Although mathematical techniques could assist in some cases, often the motion interference and heart‐rate signals overlap so much that the two are impossible to separate.^[^ ^25^ ^]^ Therefore, there is a need for improved wearable heart rate monitors. An alternative to optical methods are sensors that detect the pulse pressure wave on the radial artery.^[^ ^24^, ^25^ ^]^ Due to its high sensitivity, the PMMA‐coated LIG pressure sensor was tested for this purpose. The LIG sensor shown in Figure 2b was fixed to the wrist using an elastic band, which enabled real‐time monitoring of the cardiac activity, as illustrated in **Figure** **7**a. The magnified view in Figure 7b clearly shows two peaks associated with blood flow. P1, the “novice wave,” is associated with cardiac shrinkage, while P2 is the “reflected wave” developed due to reflection from a peripheral blood artery. The radial artery augmentation index (AI), a determinant of the hardness of human blood, can be deduced using the obtained peaks

(3) $$AI=\frac{P2}{P1}\times 100\%$$

From the signal shown in Figure 7b, AI ≅ 69%, which is a typical value for a healthy female.^[^ ^26^ ^]^ Figure 7c shows the measurement over a duration of 10 s, from which a pulse rate of 84 beats per minute can be extracted.

### 4.3 Gait Analysis Sensors

**Figure 8:**
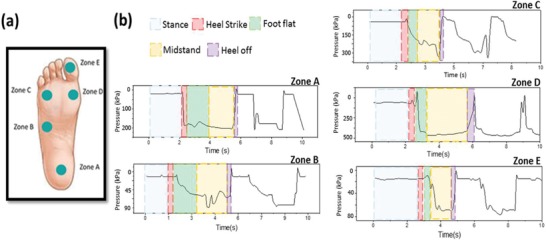
a) Arrangement of LIG sensors on five zones for plantar pressure dynamic measurements. b) Signals obtained from the right foot of an able‐bodied subject during a stride.

The foot is the terminal link of the kinematic chain in human locomotion that experiences daily repetitive stresses from bearing the weight of the body. Gait analysis has become an important tool for comprehensive physical examination, joint kinematics, motion and muscle assessment, and treatments of deficiencies in patients' limbs.^[^ ^27^ ^]^ Implementing a pressure sensor on the foot for gait analysis requires robust yet flexible, imperceptible, and lightweight sensors to avoid interfering with the gait. The LIG pressure sensors satisfy these requirements. A number of physical parameters were deduced from the pressure distribution of plantar pressure during gait analysis. We prepared five LIG sensors with dimensions shown in Figure 2b and attached them to the right foot of a test subject (55 kg in weight, and 1.60 m in height) using nylon stockings. The sensors were used to measure the pressure at five different zones on the subject's foot: the heel (Zone A), lateral arch (Zone B), first metatarsal head (Zone C), lateral metatarsal (Zone D), and hallux (Zone E), as shown in the Figure 6a. The signals in **Figure** **8**b were recorded during several full gait cycles at a sampling rate of 100 Hz, where the fundamental five stages of a full cycle are highlighted (stance, heel off, foot flat, mid‐stance, and heel off). The amplitude at different time points is associated with the foot fall on and off the ground during the walk. The highest variation of ≈470 kPa was measured at the lateral metatarsal (Zone D), while the hallux experienced the least pressure on the foot in response to walking with a value ≈84 kPa

### 4.4 Tactile Sensors

**Figure 9:**
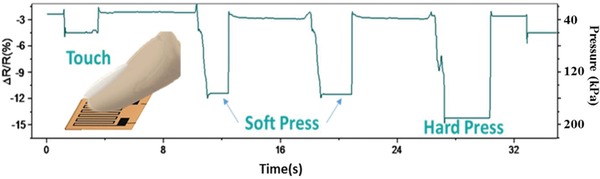
The relative resistance variation induced by touch from the index finger.

Measuring the intensity of touch is the realm of tactile sensors, which have attracted a lot of interest over the past years for robotic, prosthetic, and consumer applications.^[^ ^27^, ^28^, ^29^ ^]^ In order to mimic the tactile sense of the human skin, a sensitivity range between 0.01 and 10 N^[^ ^30^ ^]^ is required, and typically, due to the shape of the structures, flexible sensors need to be employed. In many cases, durability is a concern, since the sensors can be exposed to a corrosive environment, liquids, and elevated temperatures. LIG sensors have both a high resistance to corrosion and an operating temperature range of up to ≈400 °C.^[^ ^16^, ^20^ ^]^ **Figure** **9** shows the response recorded from an LIG tactile sensor (structure as shown in Figure 2a) in response to being pushed with the index finger by a touch, softly pressed, and hard pressed. The output increased immediately at the moment of contact of the index finger and returned to the initial values after release.

## 5 Conclusion

Using LIG, versatile and reliable pressure sensors on flexible substrates have been developed with a wide pressure range. The facile, one‐step fabrication process enables laser printing of the sensors, which is an inexpensive and versatile way for patterning graphene films in a solid state. It also allows for simple tuning of geometry, sizes, and shapes with different sensitivities and dynamic ranges. The pressure response resulted from the deformation of the conductive porous graphene structure, leading to changes in resistance, while a coating material serves as a protection from sensor damage and electrical shunting. LIG pressure sensors provide a large pressure range of at least 20 MPa with a negative relationship between pressure and relative change in resistance. Benefiting from the versatility of the laser printing technology, the size of the sensor can be easily tailored. It could be further reduced to allow integration of the sensor, for example, to create a platform for conductivity, temperature, and depth measurements for small‐sized marine animals. The PMMA‐coated sensors offer an excellent sensitivity of 1.23 × 10^−3^ kPa, a low detection limit of 10 Pa, and excellent cycling stability. Biocompatibility of the LIG sensors was demonstrated using viability and the fluorescence staining methods. The pressure sensors were utilized in several applications to demonstrate their versatility for underwater operation, as a heart rate monitor, for gait analysis, and as tactile sensor. Also, attached to the human body, the wearable sensors continued operation at large bending ranges. Due to the straight forward fabrication process, the developed sensors can be easily tailored for different applications, while also providing a route for mass production.

## Conflict of Interest

The authors declare no conflict of interest.

## Supporting information

Supporting Information

Supplemental Movie 1
