# Supplementary material for: Laser‐Printed, Flexible Graphene Pressure Sensors
Source: Glob Chall. 2020 Mar 11;4(4):2000001. doi: 10.1002/gch2.202000001 (PMC7117846; doi:10.1002/gch2.202000001)
Supplement: Supplementary file 1 — Supporting Information [file GCH2-4-2000001-s001.pdf]

Copyright WILEY-VCH Verlag GmbH & Co. KGaA, 69469 Weinheim, Germany, 2020.

Global Challenges
Open Access

Supporting Information

for *Global Challenges,* DOI: 10.1002/gch2.202000001

Laser-Printed, Flexible Graphene Pressure Sensors

*Altynay Kaidarova, Nouf Alsharif, Barbara Nicoly M. Oliveira, Marco Marengo, Nathan R. Geraldi, Carlos M. Duarte, and Jurgen Kosel**

Laser printed flexible graphene pressure sensors

Copyright WILEY-VCH Verlag GmbH & Co. KGaA, 69469 Weinheim, Germany, 2020.

Supporting Information

*Altynay Kaidarova, Nouf Alsharif, Barbara Nicoly M. Oliveira Marco Marengo, Nathan R. Geraldi, Carlos.M. Duarte, Jurgen Kosel **

Supporting information-1

Cell culture

HCT 116 (ATCC CCL247) cells were cultured in McCoy's medium 5A 1× with a l-glutamine with 10% fetal bovine serum and 1% penicillin-streptomycin. For detaching and counting the cells StemPro Accutase and trypan blue have been used, respectively. The cells were grown inside of a 37°C, humidified incubator with 5% CO2. All of the supplements and the media were bought from GIBCO life technologies.

Cell viability tests

The sensor was placed on 48-well plate (for AlamarBlue assay) and 6-well plate (for confocal) and washed three times with ethanol followed by three times with 0.01mM PBS and three times with McCoy's medium. Then, 700 000 cells were seeded on top of the sensor for 24 h.

For confocal imaging: the culture medium was removed after 24 h and exchanged with 0.01M HBSS (Gibco™ HBSS without Calcium, Magnesium or Phenol Red) and then the cells were stained for 20 min at room temperature with LIVE/DEAD® Viability/Cytotoxicity Kit (Molecular Probes™; Eugene Oregon, USA). After that, the samples washed with HBSS and investigated immediately under the confocal.

AlamarBlue assay: the culture medium was removed after 24 h and exchanged with a fresh one (200 μl) and then AlamarBlue cell viability assay (Cat. No. DAL1025) was used. Based on the vendor's protocol, 22μl of AlamarBlue reagent was added directly to the well and

incubated for 2h at 37 °C. To analyze the data, the absorbance Bio-Rad microplate reader was used at 570 nm. Both experiments were done in six replicates.

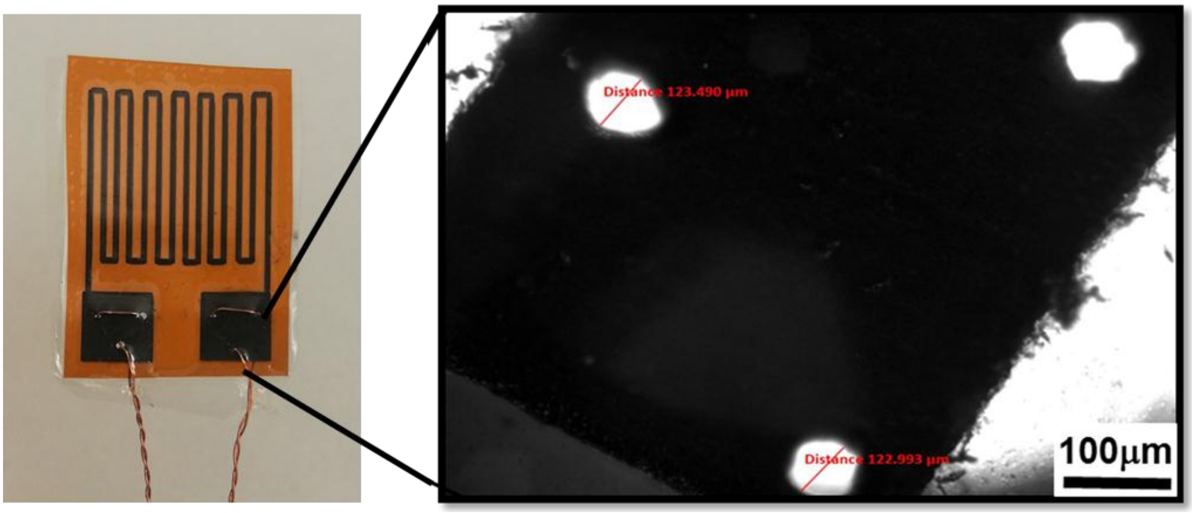

Figure S1. Mechanical contacts with LIG pressure sensor that do not involve any soldering, conductive metal depositions or riveting**.**

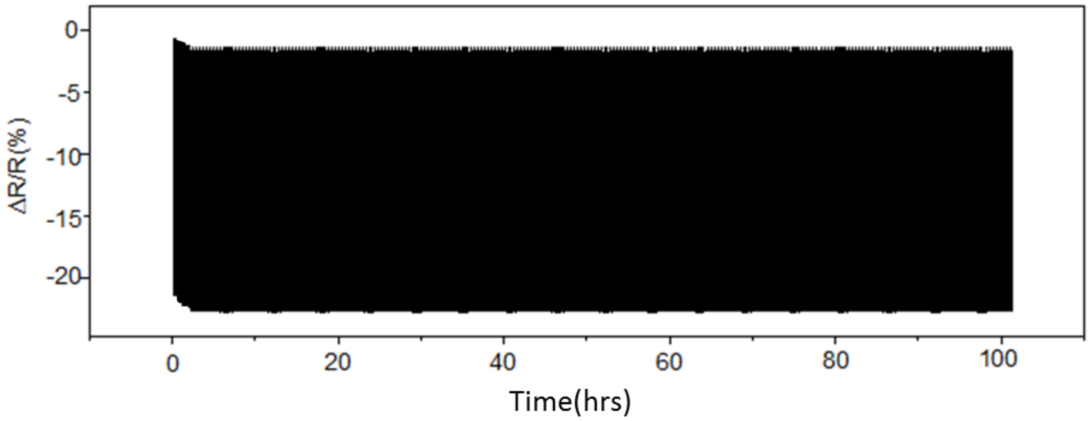

Figure S2. Long-term cycling test applying 2 kN for 15000 cycles.

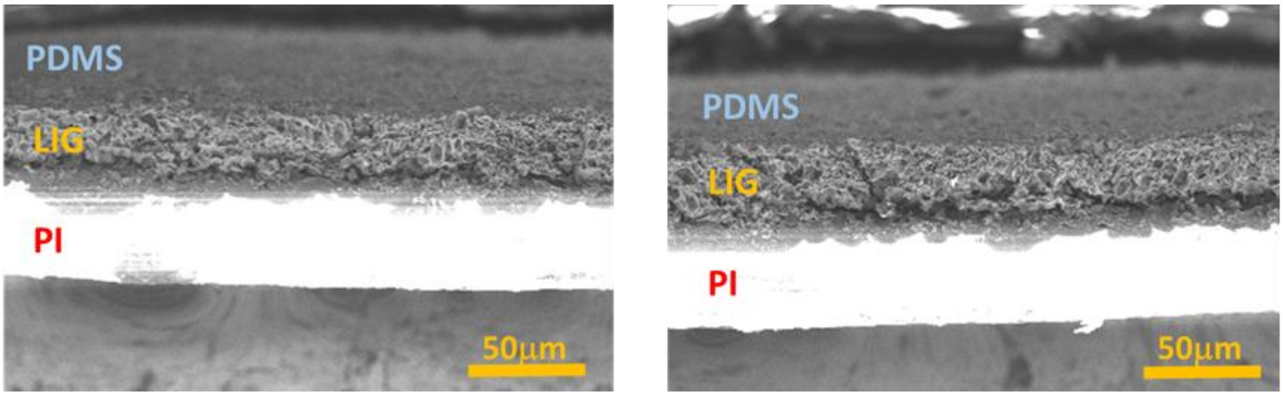

Figure S3. Cross-section of the LIG a) before and b) after exposing it to high pressures of 20 MPa.
